# Divergence and convergence of gut microbiomes of wild insect pollinators

**DOI:** 10.1128/mbio.01270-23

**Published:** 2023-07-28

**Authors:** Jilian Li, Logan Sauers, Daohua Zhuang, Haiqing Ren, Jun Guo, Liuhao Wang, Mingsheng Zhuang, Yulong Guo, Zhengyi Zhang, Jie Wu, Jun Yao, Huipeng Yang, Jiaxing Huang, Chengrui Wang, Qinghui Lin, Zhigang Zhang, Ben M. Sadd

**Affiliations:** 1 State Key Laboratory of Resource Insects, Institute of Apicultural Research, Chinese Academy of Agricultural Science, Beijing, China., Beijing, China; 2 School of Biological Sciences, Illinois State University, Normal, Illinois, USA; 3 State Key Laboratory for Conservation and Utilization of Bio-Resources in Yunnan, School of Life Sciences, Yunnan University, Kunming, Yunnan, China; 4 Faculty of Life Science and Technology, Kunming University of Science and Technology, Kunming, Yunnan, China; 5 College of Resources and Environmental Sciences, Henan Institute of Science and Technology, Xinxiang, Henan, China; 6 Shanghai Suosheng Biotechnology Co., Ltd., Shanghai, China; 7 Computer Network Information Center, Chinese Academy of Sciences, Beijing, China; 8 State Key Laboratory of Genetic Resources and Evolution, Laboratory of Evolutionary & Functional Genomics, Kunming Institute of Zoology, Chinese Academy of Sciences, Kunming, Yunnan, China; University of Hawaii at Manoa, Honolulu, Hawaii, USA; Carnegie Institution for Science, Washington, DC, USA

**Keywords:** insect pollinator, microbiota, microbiomes, symbionts, Orbaceae, *Gilliamella*

## Abstract

Pollination services provided by wild insect pollinators are critical to natural ecosystems and crops around the world. There is an increasing appreciation that the gut microbiota of these insects influences their health and consequently their services. However, pollinator gut microbiota studies have focused on well-described social bees, but rarely include other, more phylogenetically divergent insect pollinators. To expand our understanding, we explored the insect pollinator microbiomes across three insect orders through two DNA sequencing approaches. First, in an exploratory 16S amplicon sequencing analysis of taxonomic community assemblages, we found lineage-specific divergences of dominant microbial genera and microbiota community composition across divergent insect pollinator genera. However, we found no evidence for a strong broad-scale phylogenetic signal, which we see for community relatedness at finer scales. Subsequently, we utilized metagenomic shotgun sequencing to obtain metagenome-assembled genomes and assess the functionality of the microbiota from pollinating flies and social wasps. We uncover a novel gut microbe from pollinating flies in the family Orbaceae that is closely related to *Gilliamella* spp. from social bees but with divergent functions. We propose this novel species be named *Candidatus Gilliamella eristali*. Further metagenomes of dominant fly and wasp microbiome members suggest that they are largely not host-insect adapted and instead may be environmentally derived. Overall, this study suggests selective processes involving ecology or physiology, or neutral processes determining microbe colonization may predominate in the turnover of lineages in insect pollinators broadly, while evolution with hosts may occur only under certain circumstances and on smaller phylogenetic scales.

**IMPORTANCE:**

Wild insect pollinators provide many key ecosystem services, and the microbes associated with these insect pollinators may influence their health. Therefore, understanding the diversity in microbiota structure and function, along with the potential mechanisms shaping the microbiota across diverse insect pollinators, is critical. Our study expands beyond existing knowledge of well-studied social bees, like honey bees, including members from other bee, wasp, butterfly, and fly pollinators. We infer ecological and evolutionary factors that may influence microbiome structure across diverse insect pollinator hosts and the functions that microbiota members may play. We highlight significant differentiation of microbiomes among diverse pollinators. Closer analysis suggests that dominant members may show varying levels of host association and functions, even in a comparison of closely related microbes found in bees and flies. This work suggests varied importance of ecological, physiological, and non-evolutionary filters in determining structure and function across largely divergent wild insect pollinator microbiomes.

## INTRODUCTION

Wild insect pollinators provide critical ecosystem services, being crucial to the maintenance of both wild and agricultural plant communities (1, 2). Although the focus is often on managed honey bees as pollinators, thousands of wild bee and other insect species provide efficient and under-valued pollination services (3 - 7). Insect species in general are undergoing significant declines (8 - 10), but there is particular concern about wild pollinator insects facing threats that could destabilize natural ecosystems (11) and limit important agricultural production (12). Therefore, studies investigating factors linked to insect pollinator health, and thus the services they provide, that extend beyond well-studied bee pollinators are needed.

Host-associated microbes and their specific functions may determine host niche specificity, survival, and fitness (13 - 15). In insects, beneficial symbiotic bacteria can play important roles in the development and health of their hosts (16 - 18). In particular, the gut microbiota of insects has received attention due to its potentially high functional importance (13 - 15). However, the associations between gut microbes and their hosts can be highly variable (19), spanning from highly specialized gut microbial communities, such as those of social bees (20, 21), to insects with transient, environmentally determined communities or hosts with few microbes resident in their guts at all (22, 23). Given their importance for interacting with their host and the environment, it is vital to understand the composition and diversity of gut microbiota and the mechanisms that could shape these communities across relevant groups of host species.

Important questions in microbial ecology remain relating to the relative contributions of host-associated and environmental factors in determining the composition of microbiomes and what mechanisms are driving microbial community structure and function (24). The microbes colonizing the gut may be determined by neutral or selective processes (25), which can depend upon stochastic processes, host ecology, or evolutionary history. Diet has been shown to be an important factor determining the gut microbiota of mammals, with similar microbes inhabiting the guts of unrelated host but with similar diets (26). However, phylogeny and hence relatedness of hosts have also been shown to have a strong effect determining similarity in composition (27). Relationships between microbial communities that recapitulate the phylogeny of their hosts are termed examples of phylosymbiosis (28). While phylosymbiosis can emerge due to vertical transmission and co-diversification of symbionts and hosts, it can also emerge from ecological and physiological filtering that establishes interactions anew each generation from environmental microbes (29). Defining the distributions of microbes and their predicted functions among host species will help to determine the ecological and evolutionary processes that may underlie the associations. Our understanding of host-associated gut microbiomes and their potential effects can benefit from investigations of similarities and differences in community membership, community structure, and predicted function across host species that share certain ecological characteristics, such as pollination.

Studies of insect pollinator gut microbiota have mainly been in the eusocial honey bees and bumble bees (30). In these species, the gut microbiota plays important roles in nutrition, detoxification, and resistance to parasite infection (13 - 31 - 31). Associated with the corbiculate Apid bees is a relatively small core set of gut bacterial symbionts, which are thought to have largely undergone coevolution with their hosts, facilitated by vertical transmission in a social setting (20). The dynamic nature of the microbiota composition over evolutionary time, with lineage turnover, is however also apparent in this Apid clade, as the core microbes *Snodgrassella* and *Gilliamella* are absent from the stingless bee genus *Melipona*, which hosts more environmental bacteria and bee-specific yeasts (32). Studies of microbiota structure and function have been rare in other insect pollinator clades, but there is also evidence of a phylogenetic signal underlying differences in the structure of butterfly microbiomes (33). Most effective insect pollinators are either partially pollinivorous, nectarivorous, or both, and such diet sharing could influence broader patterns of microbiome and insect pollinator host associations, as it is known that diet can have important links to the gut microbiota structure and function (34). Despite an increased appreciation for diverse insect pollinators, studies documenting broad-scale patterns of gut microbiome and host associations that may be suggestive of general patterns driving host microbial community composition and functioning are warranted.

In this study, using high-throughput sequencing of 16S rRNA amplicons, we investigated the associated gut microbiomes across a wide phylogenetic representation of insect pollinators from China, spanning three holometabolous insect orders (Diptera, Lepidoptera, and Hymenoptera). To derive patterns of community structure and infer potential mechanisms shaping the gut microbial communities, from this approach, we determined: (i) bacterial genera colonization with each analyzed host genus, (ii) patterns of microbiome diversity within (alpha diversity) and between (beta diversity) the host insect pollinator genera, and (iii) if a broad phylogenetic signal underlies differences in the community composition across host genera signifying broad-scale phylosymbiosis. Additionally, utilizing metagenomic shotgun sequencing, we report three high-quality metagenome-assembled genomes (MAGs) for dominant microbial community members associated with the pollinator fly *Eristalis tenax* and the wasp *Vespa bicolor*, and infer functions based on their gene repertoires. These species were picked as they possessed clear, representative community members from the exploratory 16S analysis and are taxonomically distant from well-studied Apidae species. Based on these MAGs, we propose that pollinating flies harbor a unique species of *Gilliamella*, which we propose to be called *Candidatus Gilliamella eristali*.

## RESULTS

The gut microbiomes of a total of 861 individuals across 34 insect pollinator species (belonging to three orders: Hymenoptera, Lepidoptera, and Diptera) and two outgroup comparison species (Hemiptera: *Aphis craccivora* and *Halyomorpha halys*) were analyzed (Table S1; Fig. S1). Species were identified by morphology and by cytochrome c oxidase subunit I (COI) gene barcoding, and unless specific phylogenetic distances based on COI sequences were calculated for analyses, phylogenetic relatedness refers to previous studies (35 - 41). Amplicon sequencing of the hypervariable V3-V4 region of the bacterial 16S rRNA was performed on individual whole-gut samples. We obtained a total of 41,656,064 high-quality reads, which were passed through the DADA2 assembly and filtering with a resulting 34,897 ± 10,024 reads (mean ± SD) per sample. This resulted in reads being assigned to 27,887 amplicon sequence variants (ASVs). After filtering ASVs with taxonomic assignments belonging to eukaryotes, chloroplast, mitochondria, or no successful assignment, 26,669 ASVs remained for further analysis. Additionally, for subsequent analyses, host genera with fewer than 10 samples were excluded. This resulted in excluding three samples each from the genera *Ceratina* and *Eristalinus*, two samples each from the genera *Eucera*, *Lasioglossum*, and *Nomia*, and one sample from the genus *Sapyga*. Thus, the family Halictidae is excluded from the analysis because it consisted of only four samples, and the family Sapygidae is excluded as it consisted only of a single sample.

### Identifying colonization of bacterial genera within hosts

**Fig 1:**
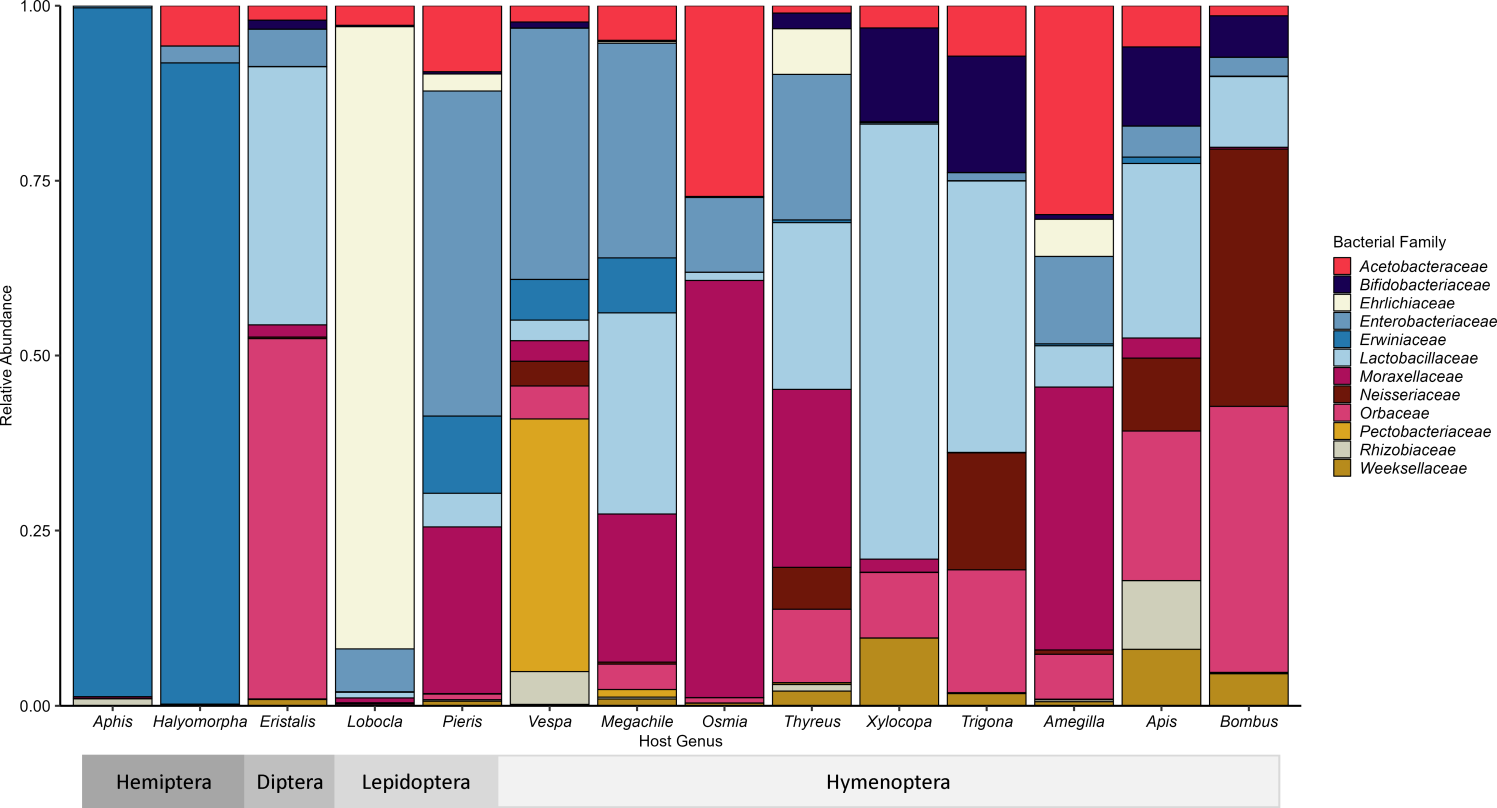
Per host genera relative abundance of microbial families with at least 1% abundance in the data set (>296,547 reads).

We identified associations between insect pollinator host genera and particular bacterial taxa, indicative of distinct ecological or evolutionary associations. Microbial ASVs were grouped into microbial genera and visualized based on relative abundance (Fig. 1). We find results that conform with some previously described relationships between hosts and their microbiomes, confirming our 16S amplicon sequencing is capturing expected native associations. First, we find low diversity within the two outgroup phloem feeding Hemiptera, with the microbial genus *Pantoea* (family *Erwiniaceae*) dominating in the host genus *Halymorpha*, which has been noted in previous work (42). Within the insect pollinators, we also observe some prior documented relationships. First, both bumble bee (*Bombus* spp.) and honey bee (*Apis* sp.) host microbiomes contain the core bacterial genera *Gilliamella*, *Snodgrassella*, and *Lactobacillus* (20, 32). The evolutionary relationships between these three genera and their Apid hosts have received substantial attention (43). Within the host genus *Osmia*, there is a large abundance of *Saccharibacter* (family *Acetobacteraceae*), within *Trigona*, there is *Bifidobacterium* and *Lactobacillus*, and within the host genus *Xylocopa*, there are significant amounts of *Apibacter*, *Bifidobacterium*, and *Lactobacillus*. These relationships have been described and documented in previous research (20, 44 - 46). Thus, we conclude that our data set accurately captures the relationships between the insect pollinator hosts sampled and their common gut communities. We uncover previously undescribed associations, including the genera *Gilliamella* and *Lactobacillus*, members of the family *Enterobacteriaceae*, such as *Cedecea*, associating with the fly genus *Eristalis*, the intra-cellular bacterium *Wolbachia* in the butterfly *Lobocla,* members of the family *Niesseriaceae* in *Trigona*, *Thyreus*, and *Vespa*, and members of the family Orbaceae in *Amegilla, Trigona, Xylocopa, Thyreus,* and *Megachile*. Additionally, we find the principally phytopathogenic genus *Lonsdalea* in samples from the Hymenopteran genus *Vespa*, which adds to previous isolated observations (47). We use a comparative genomics approach to further analyze the *Eristalis-*associated *Cedecea* and *Gilliamella* and *Vespa-*associated *Lonsdalea* to elucidate their possible functions and the nature of their relationships with the host taxa.

### Alpha and beta diversity of the pollinator microbiomes

**Fig 2:**
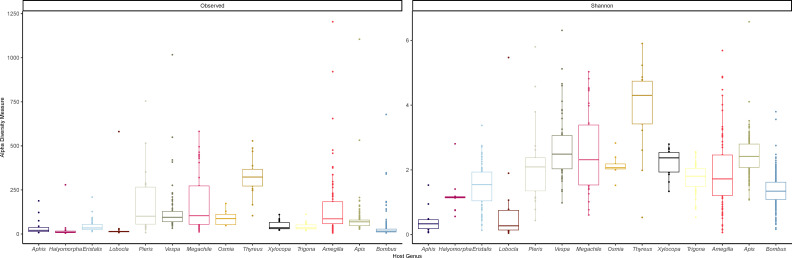
Observed species richness and Shannon alpha diversity index of gut communities for each host genus. Bars represent the medians, and the boxes show the interquartile ranges. Whiskers are the upper and lower values, with outliers shown as individual data points beyond these.

**Fig 3:**
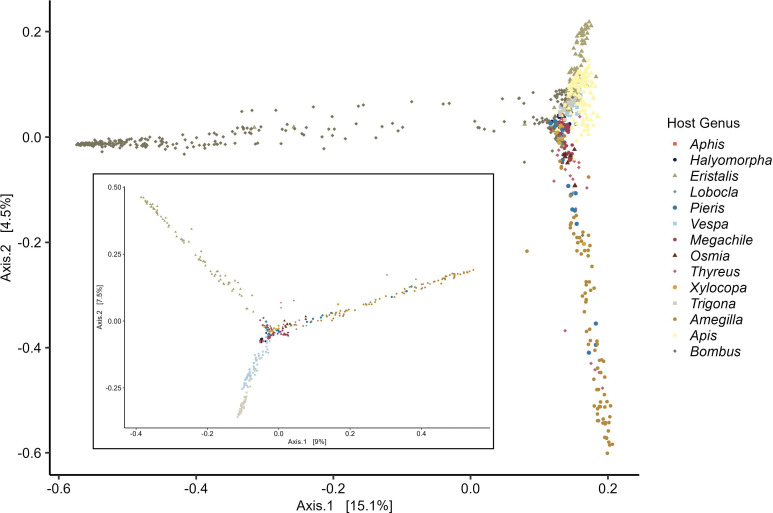
Ordination plot of the Bray-Curtis beta diversity distance among individual host microbiomes. Host genera are represented by shape and color combinations. The inset represents the same but with the genera *Apis* and *Bombus* removed.

Alpha diversity measures differed across the insect pollinator genera, and host genus explains a significant proportion of the variation in gut microbiome structure. We calculated the richness for each host genera in addition to the Shannon index. We find significant differences (*P* < 0.001) in both of these metrics across host genera (Fig. 2). *Thyreus* had the highest Shannon index, while *Aphis* and *Halymorpha* both had the lowest index values. The richness from the two Hemipterans and also from *Apis*, *Bombus*, and *Xylocopa* is low compared to many of the other genera, demonstrating that the gut microbial communities of individuals from these genera are dominated by a few abundant microbial genera. The low alpha diversity measures for these species are supported by previous work demonstrating the core conserved gut communities of these genera. Patterns of beta diversity among the genera were assessed using Bray-Curtis distances. A permutational analysis of variance (PERMANOVA) was performed on the distances between host genera and finds that there is significant partitioning of the distance variance across host genera (*P* = 0.001). The distances between samples was visualized with an ordination plot which explained 19.6% of the variation and clearing shows clustering based on host genera (Fig. 3). The pattern of structure based on host genera still holds true even with the removal of the *Apis* and *Bombus* samples (Fig. 3 inset).

### Investigating association between microbiota structure and host genetic distance

We find that gut microbiome structure is significantly determined by host genus as shown above, and our data show known and new-described relationships between certain related insect pollinators and bacterial taxa, for example, the well-established colonization of the bacterial genera *Gilliamella*, *Snodgrassella*, and *Lactobacillus* in Apid bees (20, 43, 48). However, across all the insect pollinator species analyzed, we do not find any evidence of a broad-scale relationship between host phylogenetic relatedness and similarity of the gut microbiome structure. A Mantel test was used to analyze the relationship between average pairwise distances between host genera and host microbial community Bray-Curtis distances (Table S2). We see no evidence for a significant relationship between these measures (*P* = 0.4807), suggesting no support for host relatedness determining the overall differences in gut microbiome structure across the divergent insect pollinators analyzed, which would be apparent through a phylogenetic signal. The relationship between host phylogenetic distance and associated gut microbiome structure was also not significant (*P* = 0.51) when comparing within only bee samples, where we have a greater phylogenetic breadth of diversity than in some prior comparisons (21, 43, 48, 49).

### Metagenome-assembled genomes

Three high quality metagenome assembled genomes (completeness >95% and contaminations below 1.5%) were obtained from the metagenomic sampling (Table 1). For wasp metagenomes, the initial assembly produced 3,532 contigs with the longest contig length of 253,125 bp and N50 of 3,311 bp before binning. The fly metagenome assembly produced 103,530 contigs with the longest contig length of 198,522 bp and an N50 of 7,840 bp before binning. After binning, a single bin was retrieved from the wasp assembly with 123 contigs, an N50 of 49,197 bp, a completeness of 96.72%, and contamination of 1.378%. This was automatically classified as *Lonsdalea britannica*. From the fly assembly, two high-quality bins were retrieved. The first bin consisted of 158 contigs with an N50 of 42,155 bp, a completeness of 99.79%, and contamination of 0.614%, which was automatically classified as a *Cedecea* species. The second fly-associated bin consisted of 80 contigs with an N50 of 22,570 bp, a completeness of 95.48%, and a contamination of 0.047%. This second bin was automatically classified as a *Gilliamella* species.

**TABLE 1:** Metagenome-assembled genome bins from the bee, fly, and wasp microbiota metagenome sequencing^*a*^

Bin	Completeness	Contamination	GC (%)	Contigs	N50	Size	Predict genes	Annotated genes	tRNAs	Predicted taxonomy
Fly.Bin.1	99.79	0.614	53	158	42,155	3,664,531	3,457	2,958	43	*Cedecea*
Fly.Bin.2	95.48	0.047	39	80	22,570	1,901,067	1,738	1,599	40	*Gilliamella apicola*
Wasp.Bin.1	96.72	1.378	55	123	49,197	3,749,826	3,324	2,977	51	*Lonsdalea britannica*

^*a*^ Completeness and contamination were calculated using the CheckM plugin for MetaWrap. Predicted open reading frames were determined with Prodigal, and annotation was done by matching against the KEGG, COG, and tRNA databases with Anvio.

The wasp-associated *Lonsdalea* genome is 3,749,826 bp in size with a guanine-cytosine (GC) content of 55.3%. This MAG contained 3,324 predicted open reading frames of which 2,977 were successfully annotated with a COG or KEGG function. Additionally, 51 tRNAs were identified covering 16 amino acids. The fly-associated *Cedecea* genome is 3,664,531 bp in size with a GC content of 53.4%. The genome is predicted to contain 3,457 genes with 2,958 genes annotated with a COG or KEGG function, with 43 identified tRNAs covering 17 amino acids. The fly-associated *Gilliamella* genome is 1,901,067 bp in size with a GC content of 39.1%. The MAG contained 1,738 predicted open reading frames with 1,599 being successfully annotated with a COG or KEGG function. A total of 40 tRNAs were identified covering 18 amino acids (Table 1).

### Phylogenetics and comparative genomics

**Fig 4:**
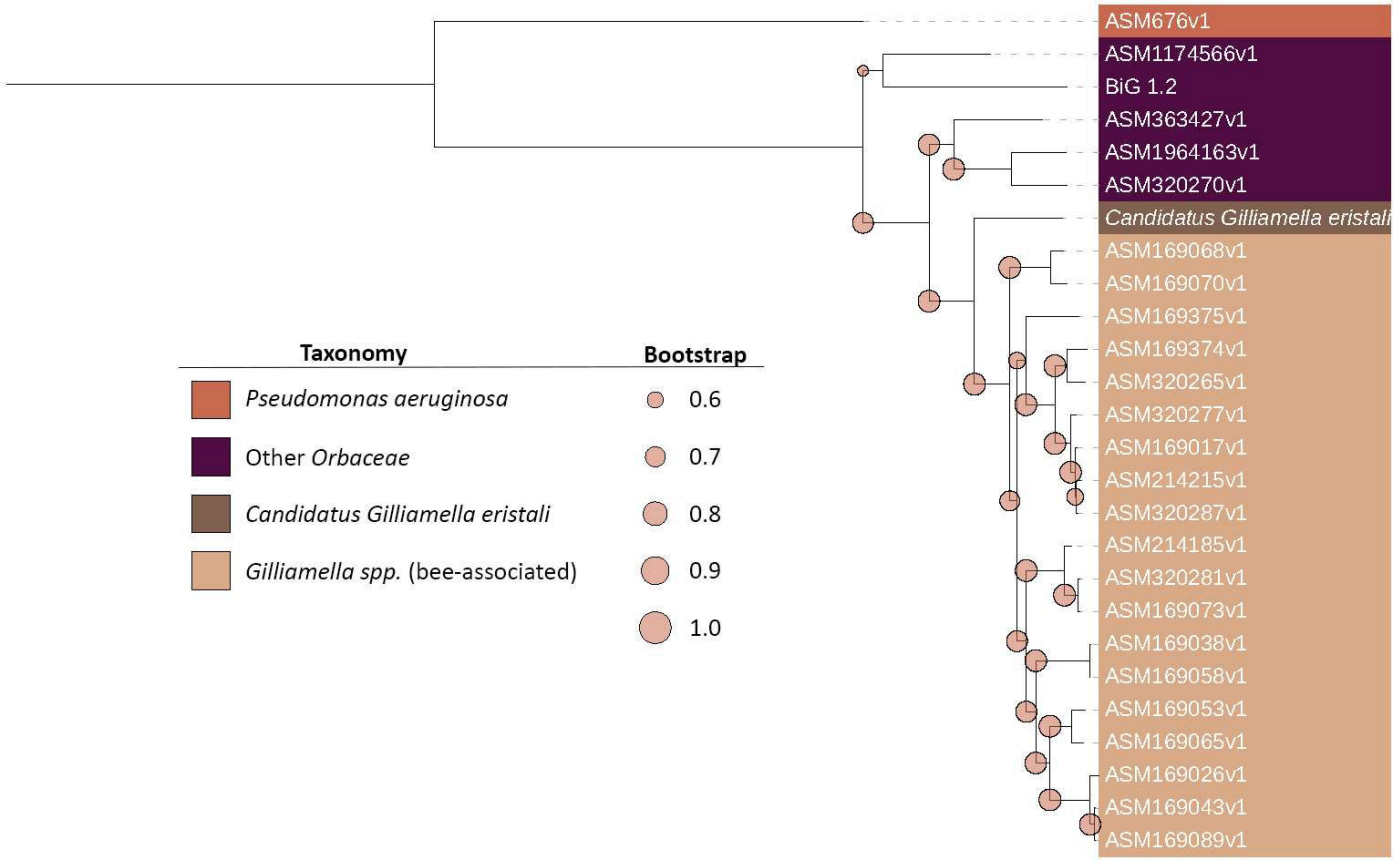
Orbaceae family phylogeny from selected core genes from the fly-associated metagenome-assembled genome assigned as *Gilliamella* sp. and existing genomes. Coloring shows different species while the branch length shows amino acid substitution rates. *Pseudomonas aeruginosa* is used as an outgroup.

To confirm the taxonomic predictions from the automatic classification and distinguish phylogenetic groupings, gene clusters were identified and phylogenies built including comparison genomes obtained for the NCBI Genome database. For the *Lonsdalea* analysis, we utilized seven genomes from four species within this genus. *Pectobacterium* sp. and *Escherichia coli* were used as outgroups. For *Cedecea*, analysis was carried out with four *Cedecea* species genomes with two *Klebsiella*. *Serratia marcescens* genomes were used as outgroups. Finally, for the fly-associated *Gilliamella* sp. we utilized Orbaceae genomes from 15 *Gilliamella apicola*, 4 *Gilliamella apis*, isolated from honeybee and bumblebee species, 2 *Frishella*, 1 *Candidatus Schmidhempelia*, 1 *Orbus*, and 1 *Zophobihabitans*, with *Pseudomonas aeruginosa* as an outgroup (Table S3). Each genome obtained was assembled at a contig level and classified as full genomes coverage. Genomes were then assembled into pan genome databases, from which a core gene set was utilized for genomic analysis. This resulted in 43 core gene clusters containing a predicted 473 genes from genomes for *Lonsdalea*, 119 core gene clusters containing a predicted 1,428 genes from the genomes for *Cedecea*, and 2 core gene clusters with 52 genes from the Orbaceae genomes. From the phylogenetic trees, we can see that our proposed *Candidatus Gilliamella eristali* clusters as a sister clade to honeybee and bumblebee *Gilliamella* species (Fig. 4). The fly-associated *Cedecea* and wasp-associated *Lonsdalea* cluster within respective clades of these genera (Fig. S2and S3), with the wasp-associated *Lonsdalea* nested within the *L. britannica* clade. Additionally, these clusterings are supported by ANIb calculations for whole genome similarities (Tables S4 through S6).

### Metabolic reconstructions

For both *Lonsdalea* and *Cedecea* samples, the metabolic functions of these bins did not significantly differ from the genome functions of closely related individuals, as determined by metabolic reconstruction and manual curation (Tables S8 and S9).

**Fig 5:**
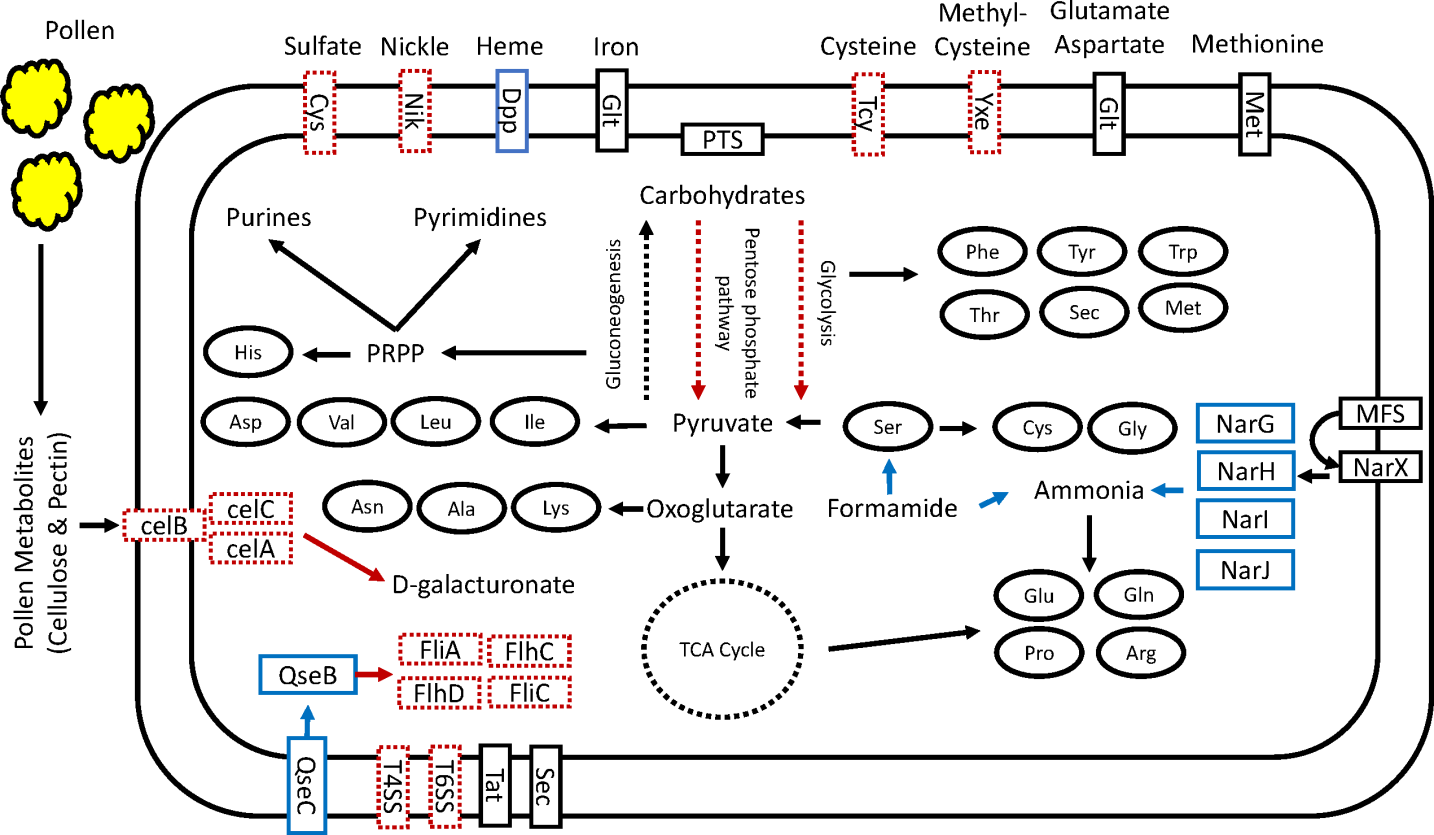
Fly-associated *Candidatus Gilliamella eristali* reconstructed metabolic pathways in comparison with other *Gilliamella* species. Solid black lines are genes/pathways shared by *Candidatus G. eristali* and other *Gilliamella* species. Dotted black lines are genes/pathways lacking across *Gilliamella* species. Dotted red lines are genes/pathways found in bee-associated *Gilliamella* but not in *Candidatus G. eristali*. Blue solid lines are genes/pathways found in *Candidatus G. eristali* but not in bee-associated *Gilliamella* species.

Substantial differences existed between our identified *Candidatus Gilliamella eristali* genome and genomes of other distinct but related *Gilliamella* species. Specifically, the *Candidatus Gilliamella eristali* lacks all genes related to cellulose permease, in addition to genes related to further degradation of pectin components found in other *Gilliamella* species. Further, *Candidatus Gilliamella eristali* lacks genes related to cysteine transport across the cell membrane, sulfate transport, and nickel transport. While marked as complete by the Anvio metabolic reconstruction tool, manual curation finds that this bacterium also lacks an essential gene for glycolysis (6-phosphofructokinase) and several genes for the pentose-phosphate pathways (L-ribokinase, L-arabinose isomerase, fructuronate reductase, L-gulonate 5-dehydrogenase) in strong contrast to the metabolic pathways of bee-associated *Gilliamella*. Interestingly, *Candidatus Gilliamella eristali* possesses a heme transporter, nitrate reductases, and a formamide conversion enzyme not found in other *Gilliamella* species (Fig. 5; Table S7). These findings are supported by the fact that many of these genes are found as being unique to *Candidatus Gilliamella eristali*, or present in several other *Gilliamella* species but not *Candidatus Gilliamella eristali* from synteny analysis (Fig. S4; Tables S10 and S11).

## DISCUSSION

Insect pollinators provide essential ecosystem services, increasing yield of agricultural crops and preserving the biodiversity of wild flowering plants (7, 10). Host insect associations with microbes can influence their health (50) and ultimately determine their ability to provide efficient pollination services (51). Accumulating evidence suggests that gut microbes associated with insects can aid their hosts in digestion, detoxification, and pathogen defense (52, 53). However, such work on associations between insect pollinators and their gut microbes is mostly constrained to a few, well-researched insect pollinators, with limited taxonomic representation. For example, most previous studies are limited to eusocial corbiculate bees, comprised of honeybees, bumblebees, and stingless bees (20). Here, we provide an exploratory examination of the gut microbial communities of diverse insect pollinators spanning the insect orders of Diptera, Lepidoptera, and Hymenoptera. From this exploratory analysis, we find significant differences in the gut community structure of these pollinators’ microbiomes, with host genus explaining a large proportion of the variation in structure between samples despite substantial variation among individuals. We find abundant indicator microbes, such as *Snodgrassella* and *Gilliamella* in *Apis* hosts, *Gilliamella* in *Eristalis*, and *Lonsdalea* in *Vespa*. Subsequently, analysis of gene repertoires suggests that these strongly associated microbes vary in apparent adaptation to the hosts, including not differing substantially from environmental-derived bacteria in some cases. This signifies that the dominance of tightly host-associated microbes seen in the Apid bees is not ubiquitous. Although similarities in microbiome structure exist between closely related genera, such as honey bees (*Apis*) and bumble bees (*Bombus*), there is no evidence of a strong relationship between gut microbiome structure and the phylogenetic relatedness of the hosts on the broad scale investigated. Surprisingly, we found a bacterium assigned as *Gilliamella* to be highly abundant not only in bees but also in the pollinator fly *Eristalis*. We used metagenomic approaches to comparatively assess the phylogenetic clustering and genetic repertoires to putatively assess function and the host-microbe relationship.

From the colonization levels of these microbes alone, we see strong divergence between taxonomically distinct insect pollinators in their gut-associated microbes, yet also some similarities, even in pollinators of different orders. The colonization abundances of microbial genera in host species and genera in our analysis supports previous findings for well-studied species (20, 32, 42 - 47), in addition to adding information about host-associated gut microbes for lesser-studied species. These include *Gilliamella* and *Lactobacillus* found to be associated with the fly genus *Eristalis*, which is interesting given that these bacteria have otherwise been associated with distinctly taxonomically unrelated bee host species in this and other studies (20). A further intriguing association is that of the dominance of *Lonsdalea* samples from the Hymenopteran *Vespa bicolor*, the black shield wasp. While acknowledged as an important pollinator (54), diverse feeding relationships in this and related species may explain this association. *Lonsdalea* is a well-described phytopathogen, particularly of trees (55), and it could be that its presence comes from feeding on tree sap that has been documented in this insect genus (56). There is another limited documentation of the *Vespa-Lonsdalea* association (47), but it is unclear, however, if the relationship could be one where the insect is acting as an alternative host (57). As the assembled metagenome of the wasp-associated *Lonsdalea* nests within a clade of plant pathogens, it seems unlikely that it is adapted to the wasp host, but raises the possibility of these functionally diverse insects acting as vectors of plant pathogens.

Alpha diversity measures also support differences in the gut microbiomes across the insect pollinators, from low richness and diversity in the butterfly *Lobocla* to high richness and diversity in the cleptoparasitic bee *Thyreus*. It is well known that the social Apid bees have a relatively small, consistent, and equal set of associated and evolutionary specialized gut bacteria (19), which is reflected in the diversity measures. The diversity of the gut bacterial communities of insects can be determined by diet, habitat, and phylogeny (58). All the assayed insect pollinators have greater diversity than the Hemipteran reference outgroups, which principally feed on plant sap. There is also the possibility that diet diversity and ecological and evolutionary interactions with microbes explain some of the differences in diversity between insect pollinators. Relatively low diversity is found in the predominantly nectar-feeding Lepidopterans. The cleptoparasitic *Thyreus* cuckoo bees’ high richness and diversity could directly or indirectly come about through their interactions with their unrelated hosts and their food stores, with female *Thyreus* laying eggs in brood cells of *Amegilla* spp. hosts before larvae emerge and consume their food provisions. Any associations between microbiome diversity and diet breadth could have an adaptive evolutionary origin, with a more diverse microbiota offering greater metabolic potential, or could result from environmental acquisition of microbes determined by diet (59). Some of the relationships identified here merit further in-depth comparative analysis to strengthen potential associations between diet and lifestyle of gut microbial community metrics.

Analysis of beta diversity, or dissimilarity of the gut bacterial communities across the insect pollinator genera, supported observations made from the relative abundances of microbes. We find a significant effect of host genera which indicates that these different pollinator species harbor distinct microbial communities. The re-emphasis that the sharing of the ecological function of pollinators, including broad diet overlap as either partial nectivores and/or pollinivores, is not sufficient to result in convergence of general microbiome community features. The distinctness of these insect pollinator host gut bacteria communities may be driven by several potential mechanisms that could act in concert or mutually exclusive on ecological and evolutionary scales. These can include neutral processes, such as the random exposure to environmental microbes, or selective processes, such as physiological filtering (25). Diet and microbiomes, as outlined above, may also be linked (25 - 34 - 34). Phylogeny and host genetic distance may also affect dissimilarity in gut microbial community composition (27, 29, 60). Phylosymbiosis, with closely related host species harboring similar symbiont communities, appears stronger for internal host-associated microbes (29). This pattern could result from vertical transmission leading to long-term coevolution, co-speciation, and co-diversification across host lineages, as proposed within social bees (20, 21), but ecological filtering by phylogenetically determined host traits may be an alternative explanation (28, 29). In fact, ecological filtering has been suggested to contribute to significant phylogenetic structuring of gut microbiomes of butterflies (33, 61). Clear close relationships between the microbiomes of the Apid bees are apparent in our data set, but across the insect pollinator host genera sampled, we find no association between pairwise genetic distances of the hosts and the dissimilarity of their gut microbial communities. Therefore, it appears that the finding that composition of the microbial communities of animals can be closely associated with host evolutionary history across wide-ranging timescales and diverse systems (60) does not extend to insect pollinators across the different orders investigated here. This could result from a neutral turnover of microbial lineages masking any phylogenetic signal between more distantly related taxa or ancestral switches in ecology, such as diet or habitat, driving selective changes in the microbiota. For example, it is likely that the ancestor of bees was predatory (62), meaning that nectarivory and pollinivory shared with distantly related insect pollinators such as the Dipteran *Eristalis* are not ancestral traits linking these lineages. As a result, host phylogenetic signals of gut microbiome structure may be restricted to finer phylogenetic scales, such as those found within Apid bees (20, 21) and butterflies (33, 61).

Following our exploratory analysis of microbial community taxonomy and diversity, we followed up with metagenomic sequencing to obtain metagenome-assembled genomes of dominant microbial community members from our sampled pollinators fly and social wasp. The pollinating fly *Eristalis* was of particular interest because we found them to possess an abundant member of the Orbaceae*,* for which we have subsequently proposed as a novel species *Candidatus Gilliamella eristali*. These flies are important non-bee pollinators (4) that also feed on nectar and pollen as adults. Wasps were selected for further analysis as social living styles have the potential to allow for more long-term host-microbe associations which may drive adaptation to the host gut environment, but their more diverse ecological roles, including diet, may also play a role in determining their microbe relationships. Host-adapted microbes have previously been characterized by smaller or reduced genome sizes, low G + C content, and unique transport, adhesion, or virulence genes allowing persistence in the gut environment (63, 64).

*Gilliamella* spp. are one of the core gut microbes of corbiculate bee, including in honey and bumble bees, and this microbe has been shown to have important functions in digestion and detoxification (24, 53). The finding of a closely related bacterium in our pollinator fly samples is intriguing and could indicate convergent membership in phylogenetically divergent but ecologically somewhat similar insect pollinator species. However, the comparative genomic analysis of the fly and bee *Gilliamella* indicates potential functional differences that may be influenced by host physiology or ecology, or the presence of other microbiota members. We found that *Gilliamella* from the bee species but not *Gilliamella* from flies have genes to digest pectin and transport cellulose into the cell. Additionally, the *Gilliamella* from bees possess genes associated with glycolysis, the pentose-phosphate pathway, flagella, sulfate, and nickel transport, and cysteine transport that are absent in *Candidatus Gilliamella eristali*. The lack of flagellar and cysteine-based proteins are especially interesting given the critical roles they have been shown to play in biofilm formation (65 - 67), and the lack of several critical genes in the oxidative portion of the pentose-phosphate pathway along with lack of 6-phosphofructokinase may point toward a more fermentative-based role in the fly gut. However, *Candidatus Gilliamella eristali* possesses genes for heme transportation, nitrate reduction, and formamide conversion not found in bee-associated *Gilliamella*. This suggests potentially important functional gene loss or gain between the *Gilliamella* isolated from flies and from bees. Gene loss could have occurred in these *Gilliamella* lineages due to evolution with their respective hosts and other co-occurring bacteria. For example, within Apid bees, cross-feeding between *Snodgrassella alvi* and *Gilliamella* has been shown to be a core feature of these communities, with a focus on iron, amino acids, and pyrimidines (48, 68). Thus, in the fly host where a co-symbiont such as *S. alvi* is absent, cysteine and siderophores may not be as readily available in the environment, and adhesion to biofilm may not be as critical for maintenance. The lack of *S. alvi* biofilm may therefore explain the lack of flagellar and cysteine acquisition genes and the presence of several heme transporters.

Metagenome-assembled genomes were also obtained for a *Lonsdalea* sp. from the *V. bicolor* wasp samples and a *Cedecea* sp. from the *E. tenax* fly samples. Here we focused on whether these genomes displayed any characteristics of host adaptation. We expected it was more likely to find signal of host adaptation-dominant wasp-associated microbes, because social living and overlapping generations of eusocial insects have the potential to foster long-term host-microbe associations. However, neither the fly nor wasp-associated genomes displayed features that are expected to reflect host adaptation. Genome length, G + C content, and predicted metabolic functionalities were similar to those of closely related bacteria species and the genomes clustered phylogenetically within other described species for both *Londsalea* and *Cedecea* isolates. Thus, while these microbes were found at relatively high abundances within the guts of these insect pollinators, it is unlikely that they are necessarily adapted to these insect hosts. In fact, as discussed previously with the case of *Lonsdalea* sp. in *V. bicolor*, they could represent phytopathogens or other environmental microbes. This suggests that host adaptation of dominant microbes may be the exception rather than the rule in insect pollinator gut communities, as highlighted by previous work (69), with rather horizontally acquired and environmentally derived communities being prevalent when considering more diverse insect pollinating hosts.

### Conclusion

Wild insect pollinators from diverse holometabolous insect orders provide key services to ecosystems. Their gut-associated microbes may influence their ecological roles and the hosts’ health, making understanding features of microbiota structure and function their underlying driving mechanisms of high importance. However, most studies have been taxonomically restricted, focusing on insect pollinator microbiomes within insect species, genera, families, or rare orders but not across more broad-scale phylogenetic ranges representing diverse insect pollinators. By doing so, we address ecological and evolutionary factors that may influence microbiome structure and function. We find that insect pollinators harbor specific microbial communities, differing in bacterial taxonomy, alpha diversity, and beta diversity. Although we uncover previously described relationships of core microbes and related hosts, such as those in the Apid bees, we find no evidence that the compositions of these microbial communities correlate with host evolutionary histories across the broader scale. Thus, we conclude that while some pollinator species may harbor vertically transmitted symbiont communities leading to phylogenetic signals, overall larger timescales, the community structure of insect pollinator microbiomes has arisen independent of host phylogenetics. Some degree of convergence between distantly related but somewhat ecologically similar taxa is suggested by the analyses and by the sharing of *Gilliamella* spp. related bacteria between distantly related bee and fly pollinators. However, comparative analysis indicates distinct functionality that could be driven by ecology and evolutionary history, and the extent of the host-microbe association. This work broadens our understanding of the microbiota of wild insect pollinators. Further, it points toward the potential importance of ecological, physiological, and non-evolutionary filters in determining microbiome structure and function when considering microbiomes on a relatively large phylogenetic scale, which calls for future in-depth comparative analyses investigating these avenues in more depth.

## MATERIALS AND METHODS

### Sample collection and processing

Samples were collected with nets in the Yunnan, Hainan, Sichuan provinces and Beijing of China from May to August 2015 (Fig. S1; Table S1). All samples were alive when captured and were then stored at –80°C. Host species identification was initially carried out by experienced field biologists with further confirmation based on analyzing the cytochrome c oxidase subunit I gene. COI sequences were first obtained from assembled contigs and then confirmed with Sanger sequencing. They were subsequently compared against the NCBI non-redundant nucleotide database and the BOLD database (http://www.boldsystems.org/) for host species identification and confirmation.

For each sample, the whole gut (including crop, midgut, ileum, and rectum) was dissected out aseptically and homogenized. This homogenate was used for DNA extraction using DNeasy Blood & Tissue kit (Qiagen, GmbH, Germany) according to the manufacturer’s instructions. DNA samples were then further purified with a Qiagen QIAquick column and eluted in 30 µL Buffer EB (Qiagen, Hilden, GmbH). The final extracts were quantified using a Qubit dsDNA broad range assay (Invitrogen, Life Technologies, Grand Island, NY, USA), and the resulting DNA samples were sent to the Shanghai Meiji for PCR amplification and sequencing.

### Amplicon and metagenomic sequencing

The hypervariable V3-V4 region of the bacterial 16S rRNA gene was amplified with the primers 341F (5′-CCTAYGGGRBGCASCAG-3′) and 806R (5′-GGACTACNNGGGTATCTAAT-3′). Twenty microliters of PCR reactions was set up with 4 µL 5 × FastPfu Buffer, 2 µL dNTPs (2.5 mM), 0.8 µL each primer, 0.4 µL FastPfu Polymerase, and template DNA (10 ng). Reactions occurred in a GeneAmp 9700 (ABI) thermocycler with 95°C for 5 min, 27 cycles of denaturation at 95°C for 30 s, annealing at 55°C for 30 s, and elongation at 72°C for 45 s, followed by an additional elongation at 72°C for 10 min. A dissociation stage was performed at the end of the run for quality control. PCR products were detected by 2% agarose gel electrophoresis, and again purified using the QIAquick Gel Extraction Kit (Qiagen). Library pools were constructed with equal amounts of each PCR product by using Truseq Nano DNA LT Sample Prep Kit (Illumina). These were amplified through paired-end sequencing on the Illumina MiSeq platform.

We performed metagenomic sequencing by selecting at random and mixing 10 intestinal samples from the same species (*V. bicolor* and *E. tenax*, respectively) or from the genus *Apis* (five samples from each of *A. cerana* and *A. dorsata*) (Table S1). The paired-end 250 bp sequencing strategy based on the Illumina MiSeq platform was adopted for metagenomic sequencing. At least 20Gbp of raw data for each mixed sample was obtained.

### Bioinformatic analysis

Amplicon sequences were processed, analyzed, and filtered using the protocol described in QIIME2 (version 2022.2) moving pictures tutorial (70, 71). Due to the arguments laid out in McMurdie and Holmes (72), we did not rarefy our data set and opted to utilize differential abundance analysis; despite this, when data were rarefied, our results remained consistent. Adapters and barcodes were removed with *cutadapt* package in QIIME2. Sequences were trimmed, filtered, assembled, and chimeras removed with QIIME2 (version 2022.2) using the DADA2 package, and were truncated at 270 bp and 200 bp for the forward and reverse sequences, respectively, as quality decreases (Fig. S2)(70, 73). The taxonomy of the ASVs was assigned using Naïve-Bayes automatic classification against the SILVA SSU database 138 full sequence database (74) with default parameters using QIIME2 (version 2022.2). Subsequent analysis was done in R using the phyloseq package, with reads associated with chloroplast, mitochondrial, and eukaryotes filtered out of the data. We then utilized the identified ASV data set and corresponding taxonomic assignments to construct the ASV count table with taxonomy data.

For metagenomic sequencing, the MetaWrap pipeline (75) was used to identify high-quality bins with above 80% completion and less than 10% contamination. First, reads were run through the quality control model, trimming adapters and poor-quality bases with Trim-galore and removing host reads with bmtagger packed into MetaWrap. Paired reads with only one read mapping to the host genome are also removed. Following quality control, the remaining reads were assembled into sample-based assemblies with metaSpades (76) and binned with Metabat2, Maxbin2, and concoct (77 - 79). The resulting bins were then refined with the MetaWrap refinement module and bin completion, and contamination estimated with CheckM (80). Bins below 80% completion and above 10% contamination were removed.

### Microbial community analysis

Based on genus abundance of gut bacteria, data were imported into R statistical software (81) using the phyloseq package (82). Phyloseq allows for community-level analysis and additional package support for analyzing microbial metagenomic data. The phyloseq allowed for comparison of relative abundances to detect microbial genera that are associated with specific host genera. Shannon alpha diversity and Bray-Curtis beta diversity indices were calculated with phyloseq and the ordinate function was used to visualize the Bray-Curtis distances. PERMANOVA from the package adonis was utilized to determine whether the host genera had a significant effect on the Bray-Curtis distances. The packages seqinr, poppr, ape, and ggTree were used to work with the host COI gene data (83 - 85). Sequences were aligned with muscle and trimmed to even lengths. The host genetic distance was then calculated, and a phylogenetic tree constructed to visualize the phylogeny and ensure the COI genes were accurately capturing true host genetic relationships. Finally, to test for correlations between host genetic distance and microbiome Bray-Curtis average distances, a pairwise matrix across all host genera was constructed. This matrix excluded the outgroup species from *Aphis* and *Halyomorpha,* as the primary focus of this study is on the insect pollinator species. These matrixes were then used to perform a Mantel test of 9,999 permutations using the R package ade (86).

### Metagenomic sequences analysis

The created metagenomic bins were imported into Anvio (87) and taxonomically identified by utilizing the MetaWrap bin classification module which automatically searches 22 single-copy core genes, and searching against the Genome Taxonomy Database to assign taxonomy to each metagenome-assembled genome. Next, open reading frames were predicted with Prodigal and annotated utilizing a DIAMOND BLASTp search against both the NCBI COGs database and KEGG KOfam database (88 - 93). External genomes for pangenomic comparisons were retrieved from the NCBI database and additional information on these genomes can be found in Table S3. With the Anvio interactive interface, for each of the three comparisons (putative *Lonsdalea*, *Gilliamella*, *Cedecea*), high-quality core genes were identified with the following search parameters: a max functional homogeneity of 0.9 and a minimum geometric homogeneity of 1.0, and present only once in every genome. These core genes were then utilized to aligned with muscle, and a PhyML phylogenetic tree with bootstrapping was constructed on the phylogeny.fr platform (94 - 96). For the Orbaceae tree, the interactive tree of life was utilized for further processing, annotation, and figure creation (97). For the other trees, ggTree was used to create supplemental figures (85). The metabolic functions of these metagenomes were then reconstructed with Anvio and complete functional pathways were determined utilizing Anvio’s predict metabolism function combined with manual curation (87).

## Data Availability

The raw sequence data reported in this paper have been deposited in the Genome Sequence Archive (98, 99) in BIG Data Center (99), Beijing Institute of Genomics (BIG), Chinese Academy of Sciences, under accession number CRA003912 that is publicly accessible. The metagenome assembled genome for Candidatus Gilliamella eristali is deposited on the NCBI under BioSample accession number SAMN36440807.

## SUPPLEMENTAL MATERIAL

Supplemental figures mbio.01270-23-s0001.docx Fig. S1 to S4 and captions to supplemental tables.

Supplemental tables mbio.01270-23-s0002.xlsx Tables S1 to S11.

ASM does not own the copyrights to Supplemental Material that may be linked to, or accessed through, an article. The authors have granted ASM a non-exclusive, world-wide license to publish the Supplemental Material files. Please contact the corresponding author directly for reuse.
