# Supplementary material for: Divergence and convergence of gut microbiomes of wild insect pollinators
Source: mBio. 2023 Jul 28;14(4):e01270-23. doi: 10.1128/mbio.01270-23 (PMC10470603; doi:10.1128/mbio.01270-23)

**Title:** **Divergence and Convergence of Gut Microbiomes of Wild Insect Pollinators**

**Jilian Li^1*#^_,_ Logan Sauers^4*#^, Daohua Zhuang^2^****^#^, Haiqing Ren^2^, Jun Guo^6^, Liuhao Wang^7^, Mingsheng Zhuang^1,5^,** **Yulong Guo ^1^, Zhengyi Zhang^1^, Jie Wu^1^, Jun Yao^1^, Huipeng**

**Yang^1^, Jiaxing Huang^1^, Chengrui Wang^8^, Qinghui Lin^8^, Zhigang Zhang^2,3*^ and Ben M. Sadd^4*^**

1. Key Laboratory of Pollinating Insect Biology of the Ministry of Agriculture, Institute of Apicultural Research, Chinese Academy of Agricultural Science, Beijing, 100093, China
2. State Key Laboratory for Conservation and Utilization of Bio-Resources in Yunnan, School of Life Sciences, Yunnan University, No.2 North Cuihu Road, Kunming, Yunnan, 650091, China
3. State Key Laboratory of Genetic Resources and Evolution, Laboratory of Evolutionary & Functional Genomics, Kunming Institute of Zoology, Chinese Academy of Sciences, Kunming 650223, Yunnan, China
4. School of Biological Sciences, Illinois State University, Normal, Illinois 61790, United States of America
5. Shanghai Suosheng Biotechnology Co., Ltd. Shanghai 201700, China.
6. Faculty of Life Science and Technology, Kunming University of Science and Technology, Kunming, Yunnan 650500, China
7. College of Resources and Environmental Sciences, Henan Institute of Science and Technology, Xinxiang, Henan 453003, China
8. Computer Network Information Center, Chinese Academy of Sciences, Beijing, 100083, China

***Correspondence:**

**Jilian Li**

Key Laboratory of Pollinating Insect Biology of the Ministry of Agriculture, Institute of Apicultural Research, Chinese Academy of Agricultural Science, Beijing, China

Email: bumblebeeljl@hotmail.com

**Or**

**Logan Sauers**

School of Biological Sciences, Illinois State University, Normal, Illinois 61790, United States of America

Email: [lsauers@ilstu.edu](mailto:lsauers@ilstu.edu)

**Ben M. Sadd**

School of Biological Sciences, Illinois State University, Normal, Illinois 61790, United States of America

Email: [bmsadd@ilstu.edu](mailto:bmsadd@ilstu.edu)

**Zhigang Zhang**

State Key Laboratory of Genetic Resources and Evolution, Laboratory of Evolutionary & Functional Genomics, Kunming Institute of Zoology, Chinese Academy of Sciences, Kunming 650223, Yunnan, China

Email: [zhangzhigang@ynu.edu.cn](mailto:zhangzhigang@ynu.edu.cn)

**Jilian Li, Daohua Zhuang and Logan Sauers contributed equally to this work.**

**Figure Legends**

**Figure S1.** Geographical locations of 861 individual samples in this study.

**Figure S2.** *Cedecea* genera phylogeny from selected core genes from the metagenome assembled genome and datamined genomes from the NCBI. Coloring shows different species while the branch length shows amino acid substitution rates. *Serratia marcescens* genomes are used as outgroups.

**Figure S3.** *Lonsdalea* genera phylogeny from selected core genes from the metagenome assembled genome and datamined genomes from the NCBI. Coloring shows different species while the branch length shows amino acid substitution rates. *Pectobacterium* and *Escherichia coli* genomes are used as outgroups.

**Figure S4.** Synteny of prodigal predicted gene clusters for *Candidatus* *Gilliamella eristali* with other *Orbaceae* genomes obtained from the NCBI. Gene clusters present are signified by black coloring while those that are absent are white. Gene clusters with orthology identified through COG of KOFAM annotations are denoted under the synteny with green coloring. Total length refers to the base pair length of the genome and is shown in the purple figure to the right of the synteny, G-C content shown in the green figure, and the number of genes per kilobase shown in the red figure.

**Table S1.** Sample information

**Table S2. A)** Average Bray-Curtis distance matrix between host genera. **B)** Host genetic distance matrix used for Mantel correlation test.

**Table S3.** Datamined genomes from the NCBI with the taxonomy, source of the cultured sample, listed genome representation, and assembly level.

**Table S4.** *Orbaceae* ANIb calculated by Anvio representing whole genome level nucleotide variations.

**Table S5.** *Cedecea* ANIb calculated by Anvio representing whole genome level nucleotide variations.

**Table S6.** *Lonsdalea* ANIb calculated by Anvio representing whole genome level nucleotide variations.

**Table S7.** *Candidatus Gilliamella eristali* metabolic predictions from Anvio. The cutoff threshold is set at the Anvio default (0.75). For further analysis each pathway was manually investigated.

**Table S8.** Metabolic predictions for the fly associated *Cedecea*. The cutoff threshold is set at the Anvio default (0.75). For further analysis each pathway was manually investigated.

**Table S9.** Metabolic predictions for the wasp associated *Lonsdalea*. The cutoff threshold is set at the Anvio default (0.75). For further analysis each pathway was manually investigated.

**Table S10.** Table of genes which are unique to *Candidatus Gilliamella eristal.* Non-*Gilliamella* genomes were excluded from this analysis.

**Table S11.** Table of genes present in other *Gilliamella* species but no present in *Canditatus Gilliamella eristali*. Non-*Gilliamella* genomes were excluded from this analysis.

**Figures.**

**Figure S1.**

**
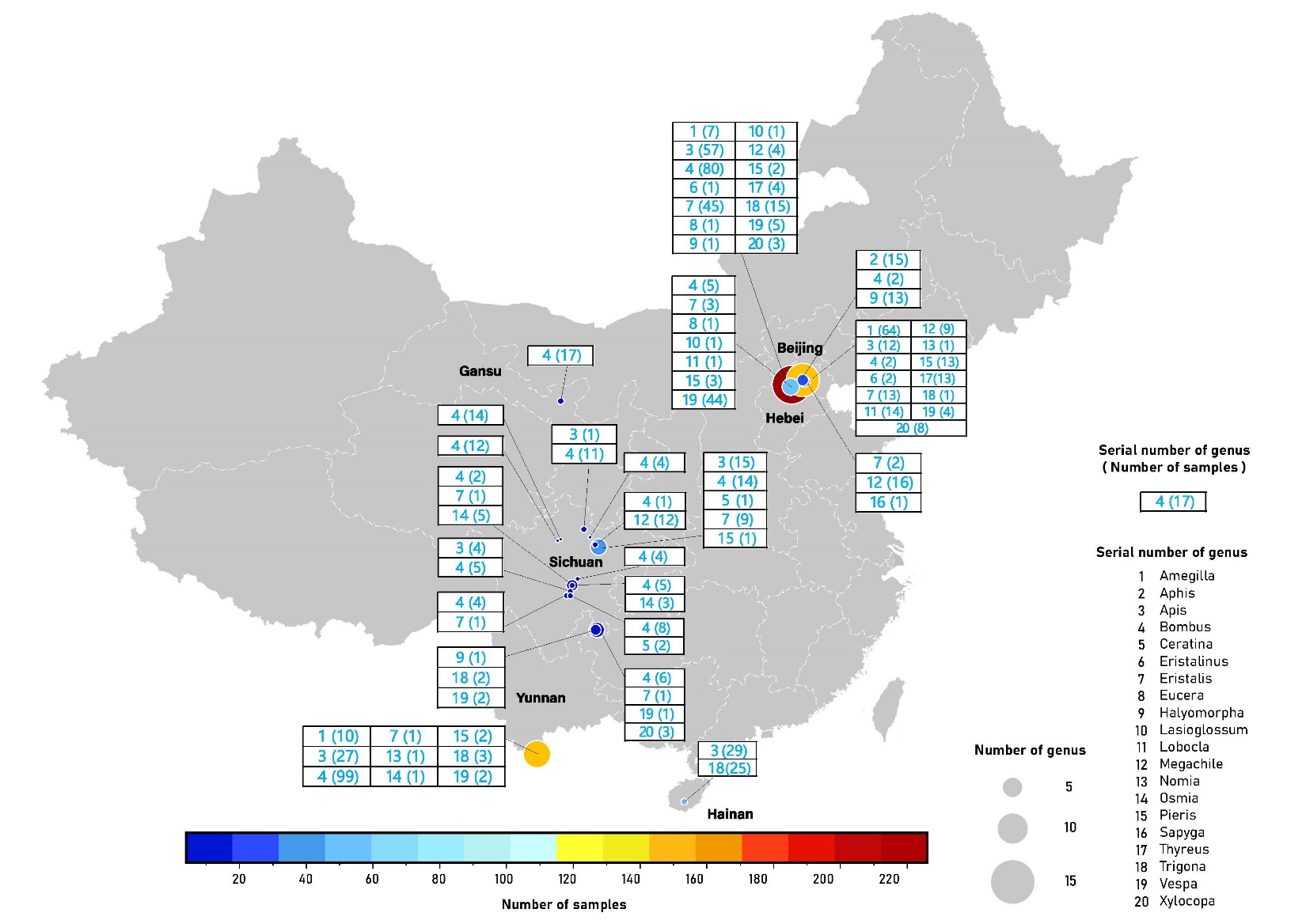
**

**Figure S2.**

**
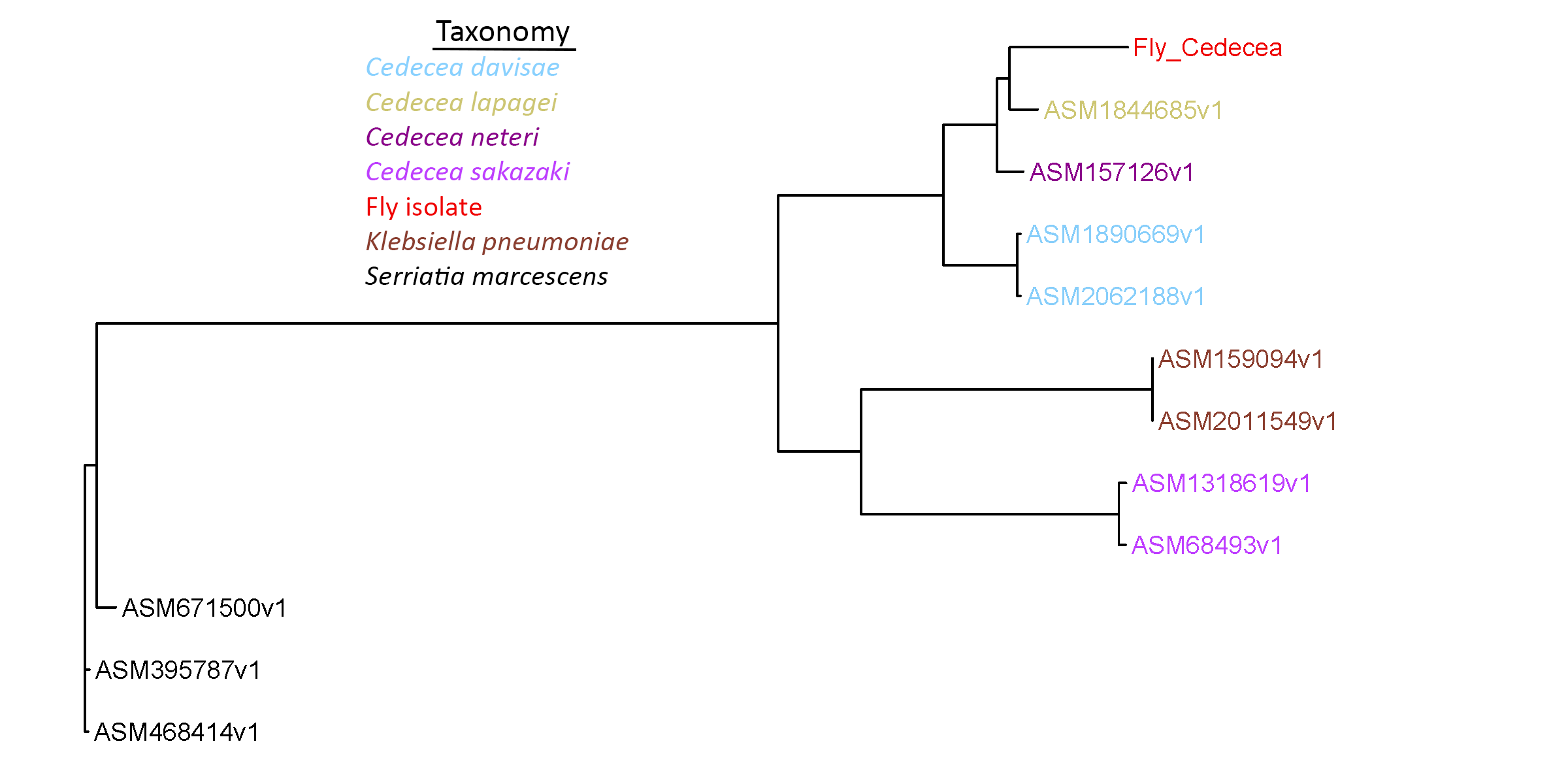
**

**Figure S3.**

**
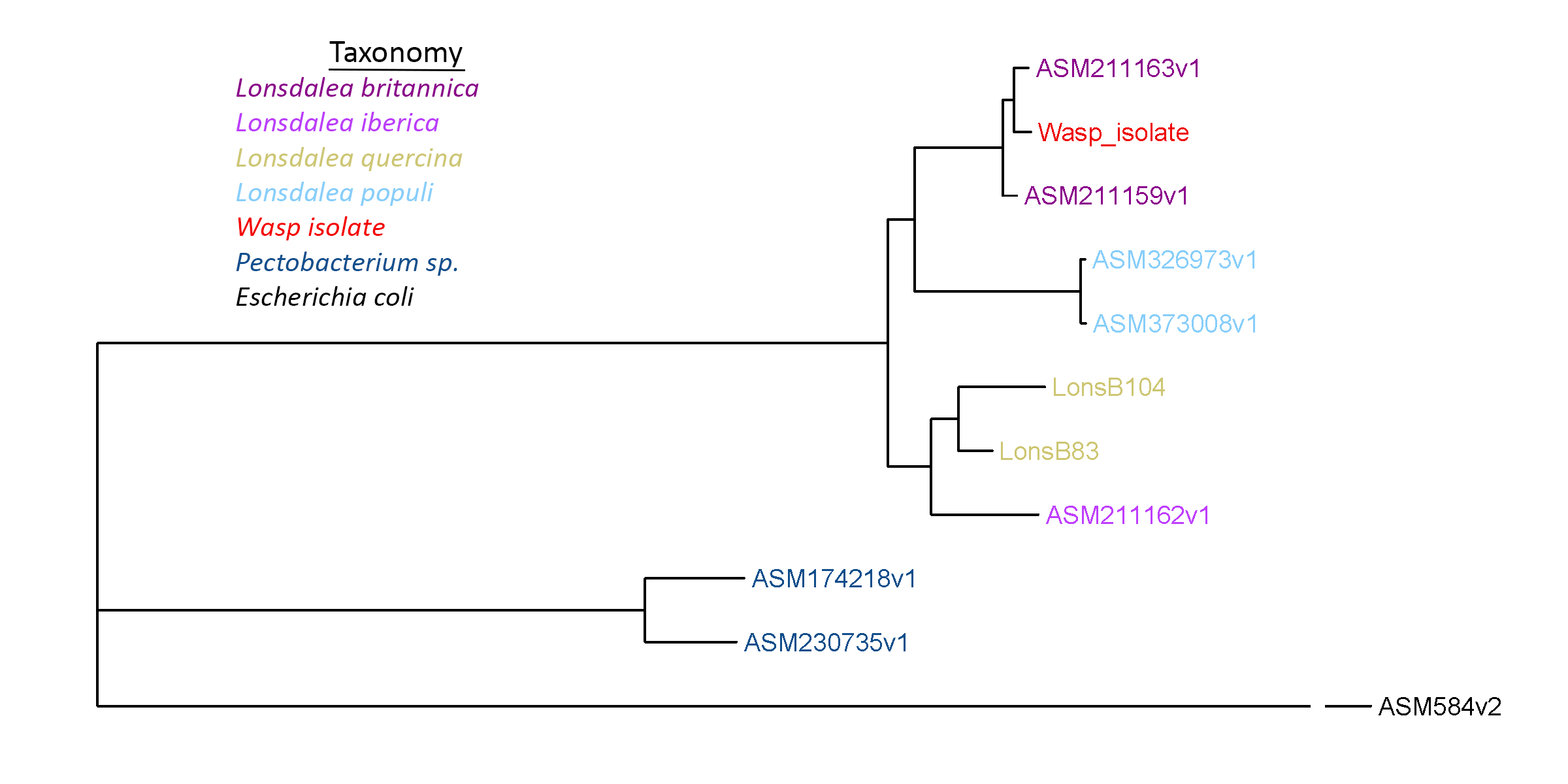
**

**Figure S4.**


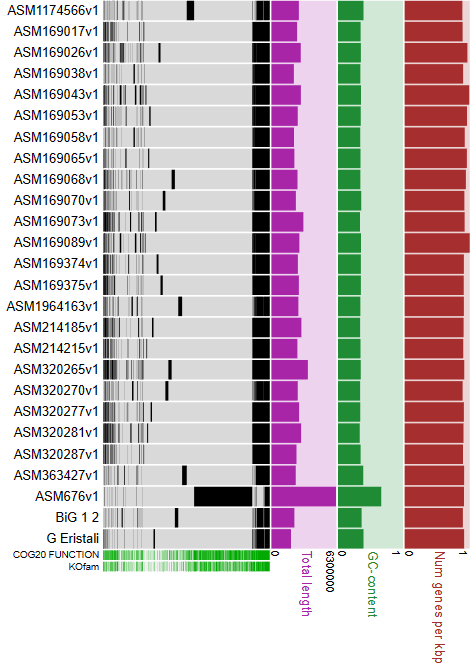

Supplement: Supplemental figures — Fig. S1 to S4 and captions to supplemental tables. [file mbio.01270-23-s0001.docx]
